# Expression of Genes for a Flavin Adenine Dinucleotide-Binding Oxidoreductase and a Methyltransferase from *Mycobacterium chlorophenolicum* Is Necessary for Biosynthesis of 10-Methyl Stearic Acid from Oleic Acid in *Escherichia coli*

**DOI:** 10.3389/fmicb.2017.02061

**Published:** 2017-10-23

**Authors:** Shuntaro Machida, Ranjith K. Bakku, Iwane Suzuki

**Affiliations:** ^1^Graduate School of Life and Environmental Sciences, University of Tsukuba, Tsukuba, Japan; ^2^Faculty of Life and Environmental Sciences, University of Tsukuba, Tsukuba, Japan

**Keywords:** branched-chain fatty acid, 10-methyl octadecanoic acid, mid-chain methyl-branched fatty acids, SAM-dependent methyltransferase, tuberculostearic acid

## Abstract

In living organisms, modified fatty acids are crucial for the functions of the cellular membranes and storage lipids where the fatty acids are esterified. Some bacteria produce a typical methyl-branched fatty acid, i.e., 10-methyl stearic acid (19:0Me10). The biosynthetic pathway of 19:0Me10 *in vivo* has not been demonstrated clearly yet. It had been speculated that 19:0Me10 is synthesized from oleic acid (18:1Δ9) by *S*-adenosyl-L-methionine-dependent methyltransfer and NADPH-dependent reduction via a methylenated intermediate, 10-methyelene octadecanoic acid. Although the recombinant methyltransferases UmaA and UfaA1 from *Mycobacterium tuberculosis* H_37_Rv synthesize 19:0Me10 from 18:1Δ9 and NADPH *in vitro*, these methyltransferases do not possess any domains functioning in the redox reaction. These findings may contradict the two-step biosynthetic pathway. We focused on novel *S*-adenosyl-L-methionine-dependent methyltransferases from *Mycobacterium chlorophenolicum* that are involved in 19:0Me10 synthesis and selected two candidate proteins, WP_048471942 and WP_048472121, by a comparative genomic analysis. However, the heterologous expression of these candidate genes in *Escherichia coli* cells did not produce 19:0Me10. We found that one of the candidate genes, WP_048472121, was collocated with another gene, WP_048472120, that encodes a protein containing a domain associated with flavin adenine dinucleotide-binding oxidoreductase activity. The co-expression of these proteins (hereafter called BfaA and BfaB, respectively) led to the biosynthesis of 19:0Me10 in *E. coli* cells via the methylenated intermediate.

## Introduction

In living organisms, modified fatty acids are crucial for the functions of the cellular membranes and storage lipids where these fatty acids are esterified (Kniazeva et al., 2004). Certain bacteria produce methylated fatty acids, such as cyclopropane fatty acids (CFAs), branched-chain fatty acids, and mycolic acids (MAs) (Akamatsu and Law, 1970; Cronan et al., 1974; Takayama et al., 2005). Some bacteria, such as *Escherichia coli* and *Lactobacillus arabinosus*, synthesize CFAs, which contain a cyclopropane ring in the acyl group. CFA synthase in *E. coli* catalyzes the modification of acyl chains to their cyclopropane derivatives through the methylation of an unsaturated bond. In this reaction, *S*-adenosyl-L-methionine (SAM) is used as a methyl donor. The enzyme acts on the *cis*-double bond at the Δ9 or Δ11 in C16 and C18 fatty acids attached to the membrane lipids (Law, 1971; Cronan et al., 1974; Grogan and Cronan, 1997; Machida et al., 2016). Branched-chain fatty acids can be classified into two types, namely *iso*- or *anteiso*-methyl-branched fatty acids and mid-chain methyl-branched fatty acids (mBFAs). The *iso*-methyl-branched fatty acids have the methyl branch on the second carbon from the methyl end of the acyl group, whilst the *anteiso*-methyl-branched fatty acids have the methyl branch on the third carbon from the methyl end. The biosynthetic pathway of these fatty acids is well-characterized in *Bacillus subtilis* (Namba et al., 1969; Oku and Kaneda, 1988). Isobutyrate, isovalerate, and 2-methylbutyrate derived from the degradation of the branched-chain amino acids, e.g., valine, leucine, and isoleucine, respectively, are thioesterified with coenzyme A to be activated and utilized as precursors of fatty acid synthesis, and then the *iso*- or *anteiso*-methyl-branched fatty acids are synthesized. As an mBFA, 10-methyl stearic acid (also called tuberculostearic acid or 10-methyl octadecanoic acid, hereafter described as 19:0Me10) is mainly known as a major component of the lipids of the tubercle bacillus (Lennarz et al., 1962). It is hypothesized that 19:0Me10 is synthesized by a two-step biosynthetic pathway (Jaureguiberry et al., 1965; Akamatsu and Law, 1970). The first step of the biosynthesis is the methylenation of oleic acid (18:1Δ9) with SAM as the methyl donor, and the resulting 10-methylene octadecanoic acid has been identified from cells of a *Corynebacterium* sp. (Couderc et al., 1991). The second step is the reduction of the methylenated fatty acid to the 10-methyl fatty acid with NADPH as the reducing agent (Akamatsu and Law, 1970). MAs are also well-known as a main component of the lipids in the envelope of mycobacteria and related species, including the genera *Mycobacterium, Nocardia, Rhodococcus*, and *Corynebacterium* (Asselineau and Asselineau, 1978; Barry et al., 1998). MAs have β-hydroxy-α-alkyl branched structures with a high molecular mass. In *Mycobacteria*, they contain approximately 60 to 90 carbons (Qureshi et al., 1978). Depending on the species, MAs contain a variety of modifications, including carbon double bonds (C = C), cyclopropane rings, and methyl branches (Takayama et al., 2005). The identification of some genes encoding the enzymes that catalyze these modifications of MAs have been reported, and among them, it has been unraveled that MmaA1 is involved in the conversion of the unsaturated double bond to the allylic methyl branch using SAM as a methyl donor (Yuan and Barry, 1996; Yuan et al., 1997).

It has been speculated that methyltransferases from *Mycobacterium* are involved in the biosynthesis of 19:0Me10. Meena et al. (2013) demonstrated the synthesis of 19:0Me10 by a recombinant protein encoded by the *umaA* gene from *Mycobacterium tuberculosis* H_37_Rv. In this study, 18:1Δ9 connected with phosphatidylcholine (PC) was ^3^H-labeled by incubation with the recombinant UmaA protein, *S*-adenosyl-[methyl ^3^H] methionine (^3^H-labeled SAM), and NADPH. Additionally, Meena and Kolattukudy (2013) also demonstrated the synthesis of 19:0Me10 by a recombinant protein encoded by the *ufaA1* gene from *M. tuberculosis* H_37_Rv. In this study, the methylester of 19:0Me10 was detected by gas chromatography (GC) with a flame-ionization detector in a mixture comprising a crude extract from *E. coli* cells expressing UfaA1, PC or phosphatidylethanolamine containing 18:1Δ9, SAM, and NADPH. Both UmaA and UfaA1 are methyltransferases that do not possess any domains functioning in the redox reaction. These findings contradict the two-step biosynthetic pathway predicted previously. In the study that focused on UmaA, ^3^H-labeled PC was separated by silica gel thin-layer chromatography and detected by fluorography. There has been no direct evidence for the synthesis of 19:0Me10 by UmaA. In the study that focused on UfaA1, the unidentified proteins in the crude extract from *E. coli* cells might have played important roles in the reduction of the methylenated intermediate.

The biosynthetic reactions of CFA, mBFA, and MA are similar processes that include the SAM-dependent methylation of the unsaturated double bond in the unsaturated fatty acids (Takayama et al., 2005). Here, we paid attention to the methyltransferase for the biosynthesis of these modified fatty acids and screened potential methyltransferase candidates for the synthesis of 19:0Me10 through a genomic comparison among various bacteria. We ascertained the existence of the modified fatty acids in the cells of those organisms via a literature survey. The candidate methyltransferases were functionally investigated *in vivo* by heterologous expression in *E. coli* cells as a host. We found that a methyltransferase and a reductase catalyze the two-step reaction for the biosynthesis of 19:0Me10.

## Materials and Methods

### Organisms and Culture Conditions

As host cells, we used two strains of *E. coli*, namely JM109 (Yanisch-Perron et al., 1985) for the construction of plasmids and Rosetta^TM^ 2 (Merck Millipore, Darmstadt, Germany) for the expression of the target genes. The cells were grown in 2 mL of Luria–Bertani (LB) medium (Bertani, 1951) at 37°C with shaking at 180 rpm. All transformants were maintained in LB medium solidified with 1.5% (w/v) Bacto^®^ agar (BD Biosciences Japan, Tokyo, Japan) in the presence of 100 μg/mL sodium ampicillin, 50 μg/mL chloramphenicol, or 50 μg/mL spectinomycin dihydrochloride pentahydrate, depending on the selection markers on the plasmids. To exogenously supply fatty acids to the culture of *E. coli* cells, 1 mM of each sodium salt of palmitoleic acid (16:1Δ9, Wako Pure Chemicals, Osaka, Japan), 18:1Δ9 (Tokyo Chemical Industry, Tokyo, Japan), linoleic acid (18:2Δ9,12, Funakoshi, Tokyo, Japan), γ-linolenic acid (18:3Δ6,9.12, Sigma-Aldrich Japan, Tokyo, Japan), α-linolenic acid (18:3Δ9,12,15, Funakoshi), or vaccenic acid (18:1Δ11, Sigma-Aldrich Japan) was added to the liquid LB medium. *Corynebacterium urealyticum* ATCC 43042 was grown on R agar^1^ with 0.5% (v/v) TWEEN 80 (Wako Pure Chemicals) and incubated at 37°C for 18 h.

### Plasmid Construction and Transformation

To express the target genes in *E. coli* heterologously, we constructed six plasmids, namely pM1942, pM2121, pFADO-4-M2121, pFADO-M2121-S, pFADO-M2121-I, and pFADO-M2121-PCYC (Supplementary Table 1), which are derivatives of an expression vector, pTCHT2031V (Ishizuka et al., 2006). Supplementary Figure 1 shows the processes for the construction of these plasmids. The plasmid pTCHT2031V contains a chloramphenicol resistance gene cassette (Cm^r^) and the *trc* promoter sequence (P*trc*). First, to replace the selection marker from the chloramphenicol resistance gene cassette with the spectinomycin resistance gene cassette (Sp^r^), we constructed a plasmid lacking the chloramphenicol resistance gene cassette, pTHT2031V, from pTCHT2031V by the polymerase chain reaction (PCR) amplification of the entire portion of pTCHT2031V except Cm^r^ using the primers pTCHT_Cm_remove_InF_F and pTCHT_Cm_remove_InF_R (Supplementary Table 2) and the In-Fusion^®^ HD cloning kit (Takara Bio, Ōtsu, Japan) for circularization. The candidate genes of the methyltransferases, WP_048471942 and WP_048472121, and a genomic fragment including two genes for the flavin adenine dinucleotide (FAD)-binding oxidoreductase, WP_048472120, and WP_048472121 (FADO-4-M2121), were amplified by PCR from the chromosomal DNA of *Mycobacterium chlorophenolicum* JCM 7439 as the template and primer sets, M1942_Nde_F/M1942_Bam_R, M2121_Nde_F/M2121_Bam_R, and FAD_Nde_F/M2121_Bam_R, respectively (Supplementary Table 2). The amplified DNA fragments were subcloned into a T-vector pMD19 simple vector (Takara Bio) to obtain the plasmids pMD-M1942, pMD-M2121, and pMD-FADO-4-M2121, and the DNA sequences of the inserts were confirmed. We then amplified a DNA fragment including the spectinomycin resistance gene cassette (Sp^r^) using pAM1146 (Tsinoremas et al., 1994) as a template. The Sp^r^ fragment digested with *Bgl*II and *BamH*I was inserted into pMD-M1942, pMD-M2121, and pMD-FADO-4-M2121 cleaved with *Bam*HI to obtain pMD-M1942S, pMD-M2121S, and pMD-FADO-4-M2121S, respectively. We chose the plasmids in which the Sp^r^ fragments were transcribed in the same orientation as the candidate genes. The fragments containing M1942, M2121, or FADO-4-M2121 and the Sp^r^ genes in these plasmids were excised by *Nde*I and *BamH*I and inserted into pTHT2031V digested with *Nde*I and *Bgl*II to obtain pM1942, pM2121, and pFADO-4-M2121. To insert the canonical Shine–Dalgarno (SD) sequence in *E. coli* between the two open reading frames, pFADO-4-M2121 was amplified using primer sets, SD_add_S_F/SD_add_S_R or SD_add_I_F/SD_add_I_R, and circularized using the In-Fusion^®^ HD cloning kit to obtain pFADO-M2121-S or pFADO-M2121-I, respectively. Finally, a homologous gene to polyketide cyclase in *M. chlorophenolicum* JCM 7439 was also amplified by PCR using the primers PCYC_inf_F and PCYC_inf_R, and inserted into pFADO-M2121-I using the In-Fusion^®^ HD cloning kit to obtain pFADO-M2121-PCYC.

The plasmid for the expression of the *umaA* gene in *E. coli* was also a derivative of an expression vector, pTCHT2031V. The *umaA* gene from *M. tuberculosis* (Meena et al., 2013) was artificially synthesized (Life Technologies Japan, Tokyo) and optimized for the codon usage of the cyanobacterium *Synechocystis* sp. PCC 6803. The Sp^r^ fragment digested with *BamH*I and *Bgl*II was inserted into pTAKN-2-UmaA and then cleaved with *Bgl*II to obtain pTAKN-2-UmaAS. The fragments containing *umaA* and the Sp^r^ genes in this plasmid were excised by *Nde*I and *Bgl*II and inserted into pTHT2031V digested with *Nde*I and *Bgl*II to obtain pUmaA.

### Fatty Acid Analysis

The profiles of fatty acids in the *E. coli* transformants were examined following the methods described in our previous publication (Kotajima et al., 2014; Machida et al., 2016). The cells were precipitated by centrifugation and re-suspended in 2 mL of methanol. The cell suspensions were then transferred to glass test tubes. After complete drying using a concentrating centrifuge (CC-105, Tomy Seiko, Tokyo, Japan), the pellet was re-suspended in 0.1 M hydrochloric acid methanolic solution (Wako Pure Chemicals). Then, the tubes were tightly capped and incubated at 100°C for 1 h to allow the saponification of the acyl-groups in lipids and conversion into fatty acid methyl esters (FAMEs). The resultant FAMEs were recovered using *n*-hexane. The hexane phases recovered were evaporated, and the residues containing FAMEs were dissolved in 100 μL of *n*-hexane.

To identify and quantify FAMEs, we applied 1 μl of the hexane solution to a GC-2014 GC equipped with a flame-ionization detector (Shimadzu, Kyoto, Japan). Helium was used as a carrier gas at a constant flow rate of 1.25 mL/min in splitless mode. A CP-Sil5 CB column (Agilent Technologies, Santa Clara, CA, United States) was used at the following temperatures: 60°C for 1.5 min, then 130°C at 20°C/min, and a further increase to 230°C at 4°C/min. To identify FAMEs of myristic acid (14:0), palmitic acid (16:0), 16:1Δ9, stearic acid (18:0), 18:1Δ9, 18:1Δ11, *cis*-9,10-methyleneoctadecanoic acid (19:1cycloΔ9), and 19:0Me10, we used a GC, GC-2010, equipped with a mass spectrometer, QP-2010 (Shimadzu). The conditions of GC were identical to those used for the FAME quantification, as described above. We confirmed the retention times and mass spectra of commercial FAME standards (Nu-Chek Prep, Elysian, MN, United States, >99%), 19:1cycloΔ9 (Santa Cruz Biotechnology, ≥98%), 18:1Δ11 (≥97%), and 19:0Me10 (Larodan Fine Chemicals, Malmö, Sweden, ≥97%).

*cis*-7-hexadecenoic acid (16:1Δ7), lactobacilic acid (19:1cycloΔ11), and *cis*-9,10-methylenehexadecanoic acid (17:1cycloΔ9), were identified by comparing with the mass spectrometry data library. *cis*-9,10-Methylene-*cis*-12-octa-decenoic acid (19:2Δ12cycloΔ9), *cis*-9,10-methylene-*cis*-12,15-octadecadienoic acid (C19:3Δ12,15cycloΔ9), and *cis*-11,12-methyleneoctadecanoic acid (19:1cycloΔ11) were identified from the mass (*m/z*) of the parent ions and the pattern of fragmentation in the GC–mass spectrometry results.

### Alignments of the Homologous Proteins and Construction of Phylogenic Tree

The protein sequences were obtained from the genetic information registered in the NCBI GenBank database ^2^. The alignment and construction of a phylogenic tree of the primary structures of the proteins was performed using ClustalX2^3^ and the phylogenic tree was drawn using Fig Tree v1.4.2^4^. The alignment and comparison of the primary sequences of the homologous proteins was performed using ClustalW^5^ and BoxShade Server^6^.

### Protein Assay

Proteins for analysis of sodium dodecyl sulfate-polyacrylamide gel electrophoresis (SDS-PAGE) were extracted from *E. coli* cells as follows: the cells were precipitated by centrifugation and re-suspended in 50 mM HEPES-NaOH (pH 7.0). The cell suspensions were treated by French press (model OS, Constant Systems, Ltd, Northants, United Kingdom) with 45 kpsi to disrupt the cells. Then cellular debris were removed by centrifugation (20,000 × *g*, 20 min, 4°C). Supernatant were stored as soluble fraction. Cellular debris was re-suspended in 50 mM HEPES and then stored as insoluble fraction. Sample buffer (4% SDS, 20% glycerol, 10% 2-mercaptoethanol, 0.004% bromophenol blue, 0.125 M Tris-HCl, pH 6.8) was added to each fraction, and incubated at 95°C for 5 min to allow the denaturation of the proteins. After applying of samples to 8% gel and electrophoresis, gel was stained with CBB (0.25% coomassie brilliant blue R-250, 40% methanol, and 7% acetic acid) for 2 h and decolorized moderately.

## Results and Discussion

### Heterologous Expression of UmaA Does Not Contribute to Synthesis of 19:0Me10 *in Vivo*

Meena et al. (2013) previously demonstrated that the recombinant protein encoded by the *umaA* gene from *M. tuberculosis* H_37_Rv produces 19:0Me10 from 18:1Δ9 attached to PC *in vitro* and the addition of NADPH to the reaction mixture stimulates the production of 19:0Me10. Based on this report, we attempted to synthesize 19:0Me10 *in vivo* by culturing *E. coli* cells expressing the *umaA* gene in a medium supplying 18:1Δ9 as an exogenous fatty acid. According to the report of Meena et al. (2013), the expression of UmaA in *E. coli* may produce 19:0Me10, although we could not observe any novel fatty acids, including 19:0Me10, in cells transformed with the plasmid pUmaA (data not shown). The entire portion of UmaA is occupied by the typical structure of an SAM-dependent methyltransferase and may not have any capable domains involved in the reduction step. There was a discrepancy between the results reported by Meena et al. (2013) and those of previous reports (Jaureguiberry et al., 1965; Akamatsu and Law, 1970; Couderc et al., 1991). To attempt to resolve this discrepancy, we searched for novel genes involved in the biosynthesis of 19:0Me10.

### Candidate Genes for Synthetic Enzymes of Branched Chain Fatty Acids

**FIGURE 1:**
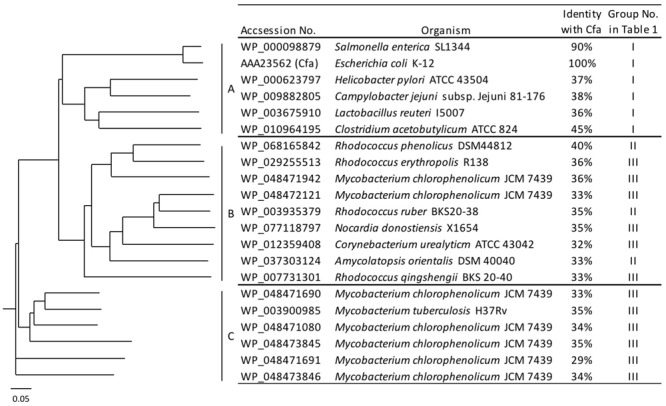
Phylogenetic tree of the *S*-adenosyl methionine (SAM)-dependent methyltransferases. Amino acid sequences of 21 SAM-dependent methyltransferases from 15 organisms were analyzed and classified into three clades: A, B, and C.

Certain bacteria produce methylated fatty acids such as CFA, mBFA, and MA. We surveyed bacteria producing methylated fatty acids from the literature. The genomic data of 15 organisms, i.e., *Salmonella enterica* subsp. serovar Typhimurium ATCC 13311 (Balamurugan, 2010), *E. coli* K-12 (McGarrity and Armstrong, 1975), *Helicobacter pylori* ATCC 43504 (Inamoto et al., 1995), *Campylobacter jejuni* subsp. Jejuni 81-176 (Asakura et al., 2016), *Lactobacillus reuteri* I5007 (Liu et al., 2014), *Clostridium acetobutylicum* ATCC 39057 (Lepage et al., 1987), *Rhodococcus ruber* KCCM 41053 (Hwang et al., 2015), *Amycolatopsis orientalis* DSM 43387 (Majumdar et al., 2006), *Rhodococcus phenolicus* DSM 44812 (Rehfuss and Urban, 2005), *M*. *chlorophenolicum* JCM 7439 (Apajalahti et al., 1986; Hagglblom et al., 1994), *Rhodococcus erythropolis* DSM 43066 (Koch et al., 1995), *Rhodococcus qingshengii* djl-6 (Xu et al., 2007)*, M. tuberculosis* H37Rv (Khuller et al., 1982), *Nocardia donostiensis* X1654 (Ercibengoa et al., 2016), and *C. urealyticum* ATCC 43042 (Couderc et al., 1991) were selected. These organisms were then categorized into three groups according to their possession of methylated fatty acids, as follows: (I) bacteria producing only CFAs, (II) bacteria producing only mBFAs, and (III) bacteria producing mBFAs and MAs (**Table 1**). It has been reported that CFA, mBFA, and MA are synthesized by a methyltransferase using SAM as a donor of the methyl group (Takayama et al., 2005). Therefore, we attempted to search for candidate methyltransferases using the *cfa* gene from *E. coli*, which is well-characterized as the gene for cyclopropane fatty acid synthase (Fagan and Palfey, 2010) and possesses an SAM-dependent methyltransferase domain, as a query sequence and the genomic sequences of the organisms listed in **Table 1** as the targets for a BLASTP search (Mount, 2007). However, the genomic sequence data of six strains, namely *S. enterica* subsp. serovar Typhimurium ATCC 13311, *C. acetobutylicum* ATCC 39057, *R. qingshengii* djl-6, *R. ruber* KCCM 41053, *R. erythropolis* DSM 43066, and *A. orientalis* DSM 43387, were not available from public databases. Therefore, the genomic DNA sequences of closely related strains, namely *S. enterica* subsp. serovar Typhimurium SL1344, *C. acetobutylicum* ATCC 824, *R. qingshengii* BKS 20-40, *R. ruber* BKS20-38, *R. erythropolis* R138, and *A. orientalis* DSM 40040, respectively, were used as surrogates. Then, we selected one potential SAM-dependent methyltransferase that had the highest homology with the query sequence from the genomic information of each organism. The genomic DNA of *M. chlorophenolicum* JCM 7439, which is categorized into group III in **Table 1**, was available from the National BioResource Center at the Institute of Physics and Chemical Research (RIKEN) and we did not know how similar the three types of methyltransferases involved in the biosynthesis of CFA, mBFA, and MA would be. Thus, while we chose only one methyltransferase from each other organism, we chose all the potential methyltransferases from *M. chlorophenolicum* JCM7439. In the chromosome of *M. chlorophenolicum*, seven SAM-dependent methyltransferases were identified (WP_048471080, WP_048471690, WP_048471691, WP_048471942, WP_048472121, WP_048473845, and WP_048473846). Then, we drew a phylogenetic tree of these protein sequences using the ClustalX2 program^3^ (**Figure 1**). The resultant phylogenic tree showed that the methyltransferases from the organisms classified into group I in **Table 1** were included in clade A, those from the organisms classified into group II were included in clade B, and those from the organisms classified into group III were included in clades B and C, suggesting that the enzymes categorized into clades A, B, and C might be related to the synthesis of CFAs, mBFAs, and MAs, respectively. Among the seven SAM-dependent methyltransferases identified in *M. chlorophenolicum*, WP_048471942 and WP_048472121 were included in clade B as shown in **Figure 1**. Thus, we hypothesized that these two proteins are potential candidate methyltransferases for the biosynthesis of the mBFAs in *M. chlorophenolicum*. To investigate their functions, we expressed each gene in *E. coli* cells using the plasmids pM1942 and pM2121 including the genes for WP_048471942 and WP_048472121, respectively, which both possessed the P*trc* promoter with the canonical SD sequence upstream of the initiation codon (Supplementary Figure 1), and analyzed their fatty acid compositions. As a result, a novel chromatography peak that was not found in the vector control strain was detected in the cells expressing WP_048472121 that were cultured in medium supplemented with 18:1Δ9 (We described the details in later section and refer in **Figures 4, 5**), but not in those expressing WP_048471942. Unfortunately, the retention time and mass spectral pattern of this peak were not identical with those of the commercially obtained mBFA standard, 19:0Me10. We then analyzed the electrophoretic separation pattern of proteins in the cells by SDS-PAGE. We found a protein band corresponding to a molecular mass of 49 kDa, which was not found in the cells of non-transformed cultures, in the lysates of the *E. coli* cells transformed with each methyltransferase gene, WP_048471942 and WP_048472121, whose predicted molecular masses are approximately 49 kDa (Supplementary Figure 2). The results indicated that the expression of the WP_048471942 and WP_048472121 methyltransferases was not sufficient to produce mBFA.

**Table 1:** Existence of each fatty acid in each organism.

Group	Organisms	CFA	mBFA	MA	Reference
	*Salmonella enterica* subsp. serovar Typhimurium ATCC 13311	+	-	-	Balamurugan, 2010
	*Escherichia coli* K-12	+	-	-	McGarrity and Armstrong, 1975
I	*Helicobacter pylori* ATCC 43504	+	-	-	Inamoto et al., 1995
	*Campylobacter jejuni* subsp. Jejuni 81-176	+	-	-	Asakura et al., 2016
	*Lactobacillus reuteri* I5007	+	-	-	Liu et al., 2014
	*Clostridium acetobutylicum* ATCC 39057	+	-	-	Lepage et al., 1987
	*Rhodococcus ruber* KCCM 41053	-	+	-	Hwang et al., 2015

	*Amycolatopsis orientalis* DSM 43387	-	+	-	Majumdar et al., 2006
II	*Rhodococcus phenolicus* DSM 44812	-	+	-	Rehfuss and Urban, 2005
	*Mycobacterium chlorophenolicum* JCM 7439	-	+	+	Apajalahti et al., 1986; Hagglblom et al., 1994
	*Rhodococcus erythropolis* DSM 43066	-	+	+	Koch et al., 1995

	*Rhodococcus qingshengii* djl-6	-	+	+	Xu et al., 2007
III	*Mycobacterium tuberculosis* H37Rv	-	+	+	Khuller et al., 1982
	*Nocardia donostiensis* X1654	-	+	+	Ercibengoa et al., 2016
	*Corynebacterium urealyticum* ATCC 43042	-	+	+	Couderc et al., 1991

*“+”, existence had been reported; “-”, existence had not been reported.*

### Two-Step Reaction for the Synthesis of mBFAs

**FIGURE 2:**
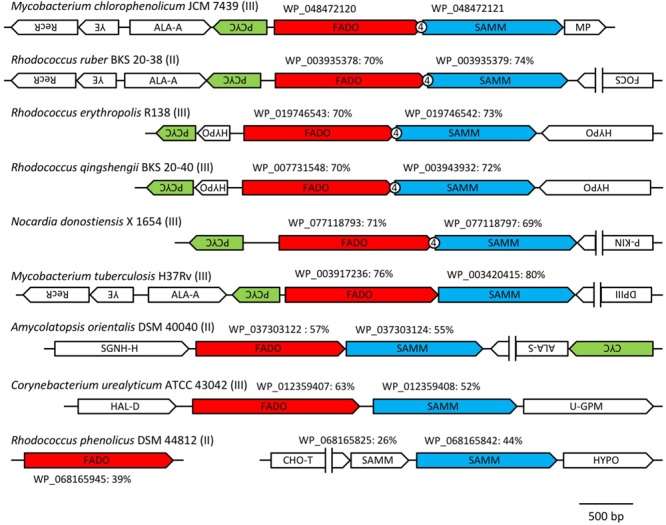
Arrangements of the orthologous genes of the FAD-binding oxidoreductase WP_048472120 and SAM-dependent methyltransferase WP_048472121 among microorganisms that produce medium branched-chain fatty acids (mBFAs). The orthologous genes of the FAD-binding oxidoreductase WP_048472120 and the SAM-dependent methyltransferase WP_048472121 were identified from the genomic sequences of *Mycobacterium chlorophenolicum* JCM 7439 (NZ_BCQY01000005), *Rhodococcus ruber* BKS 20-38 (NZ_AOEX01000025), *Rhodococcus erythropolis* R138 (NZ_CP007255), *Rhodococcus qingshengii* BKS 20-40 (NZ_AODN01000039), *Nocardia donostiensis* X 1654 (NZ_MUKP01000048), *Mycobacterium tuberculosis* H37Rv (NC_018143), *Amycolatopsis orientalis* DSM 40040 (NZ_ASJB01000001), *Corynebacterium urealyticum* ATCC 43042 (NC_010545), and *Rhodococcus phenolicus* DSM 44812 (NZ_LRRH01000116). The genes located near the orthologs of WP_048472121 were drawn schematically. The directions of the arrows indicate the orientation of transcription of the genes. The number “4” in the circle indicates the overlap of four base pairs between the two tandem genes. The group number in **Table 1** is shown next to the name of strain. The accession number of each FAD-binding oxidoreductase and SAM-dependent methyltransferase is shown above the arrow indicating each gene. The percentage values indicate the identities of the protein sequences with WP_08472120 or WP_048472121. RecR, recombination protein; YE, YbaB/Ebf family (Jutras et al., 2012); ALA-A, *N*-acetylmuramoyl-L-alanine amidase; PCYC, polyketide cyclase; FADO, FAD-binding oxidoreductase; SAMM, SAM-dependent methyltransferase; MP, membrane protein; FOCS, fatty oxidation complex subunit α; P-KIN, protein kinase; SGNH-H, SGNH hydrolase; ALA-S, acetolactate synthase; CYC, cyclase; HAL-D, haloacid dehalogenase; U-GPM, UDP-galactopyranose mutase; CHO-T, choline transporter; HYPO, hypothetical protein; DPIII, DNA polymerase III.

**FIGURE 3:**
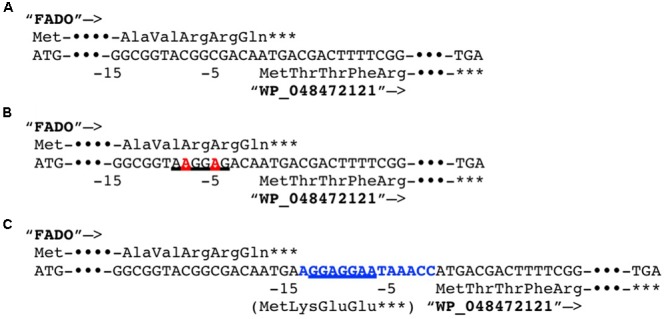
Modification of the Shine–Dalgarno (SD) sequence upstream of the coding sequence of the methyltransferase WP_048472121. **(A)** The native genomic sequences encoding the FAD-binding oxidoreductase (FADO) WP_08472120 and the SAM-dependent methyltransferase WP_048472121 was shown. **(B)** The genomic sequences substituted the two nucleotides in upstream of WP_048472121 without alteration of the encoded polypeptide sequence was shown. The substituted nucleotides are shown by a bold, red-colored font. **(C)** The genomic sequences inserted 14 nucleotides including the canonical SD sequence from *Escherichia coli* between FADO and WP_048472121. The inserted nucleotides are shown by a bold, blue-colored font. The translated amino acid sequences are shown. ^∗∗∗^Indicates the stop codons. –15 and –5 indicate positions from the translational start codon of WP_048472121. Putative SD sequences were underlined.

In a previous study, it was reported that biosynthetic pathway of mBFAs consists of a two-step reaction that includes a methylenation reaction using SAM as a donor of the methylene group to synthesize intermediates and a reduction reaction using NADPH as a cofactor to produce the saturated methyl-branched fatty acid (Oku and Kaneda, 1988). The SAM-binding site and methylase domain occupy the entirety of both the WP_048471942 and WP_048472121 proteins and they do not seem to possess any domains associated with redox reactions. Thus, we anticipated that a gene encoding another protein that catalyzes the reduction reaction might exist elsewhere in the *M. chlorophenolicum* genome. We performed a comparative genomic analysis of the structures of the genes for WP_048471942 and WP_048472121 with sequences available in public databases and found that a gene for a protein of unknown function, which is annotated as “an FAD-binding oxidoreductase” (WP_048472120), was located upstream of the gene for WP_048472121. The termination codon of the gene for the FAD-binding oxidoreductase-like protein and the initiation codon of the gene for WP_048472121 overlapped by four base pairs. Therefore, it seems possible that these genes are co-transcribed as an operon from the chromosome in *M. chlorophenolicum*, in which case the two proteins are more likely to be involved in the same reaction. Furthermore, the orthologous genes were also conserved in the genomic sequences of the organisms categorized into clades B and C as shown in **Figure 1**, and the genes in *R. ruber* BKS 20-38 *R. erythropolis* R138, *R. qingshengii* BKS 20-40, and *N. donostiensis* X 1654 also overlapped by four base pairs, like those in *M. chlorophenolicum* JCM 7439 (**Figure 2**). To investigate whether the FAD-binding oxidoreductase-like protein and the SAM methyltransferase are involved in mBFA synthesis, we constructed *E. coli* transformants to express both genes using the plasmid pFADO-4-M2121 (Supplementary Figure 1). However, we could not detect the synthesis of mBFA. Then, we analyzed the expression of the recombinant proteins by SDS-PAGE. We found a 52 kDa band corresponding to the FAD-binding oxidoreductase-like protein, but no 49 kDa band for WP_048472121 methyltransferase, in the lysates of the cells transformed with the two overlapped genes (data not shown). Then, we looked at the upstream sequence of the WP_048472121 gene (-15)-GGCGGTACGGC-(-5) and found that the sequence was somewhat different from the canonical SD sequence in *E. coli*, i.e., (-15)-AAGGAGGAATA-(-5) (**Figure 3A**). We expected that this sequence was not recognized as an SD sequence in *E. coli*. Therefore, the sequence upstream of WP_048472121 was modified by two approaches. First, the sequence was modified by substitution of the upstream nucleotide sequences without changing the encoding amino acids and the four overlapping base pairs to produce the plasmid pFADO-M2121-S (**Figure 3B**). Second, the sequence was modified by the insertion of the SD sequence with the removal of the overlap between the genes without changing the nucleotide sequence of the gene for FAD-binding oxidoreductase-like protein and creating a termination codon from the overlapped ATG to produce the plasmid pFADO-M2121-I (**Figure 3C**). The two resultant plasmids were then transformed into the *E. coli* cells. The results of an analysis of fatty acid composition showed that the cells expressing both types of modified genes accumulated a novel mBFA, 19:0Me10, when 18:1D9 was added into the culture as an exogenous fatty acid. Note that only the results for the cells transformed with the plasmid pFADO-M2121-I are shown in **Table 2**. And by the SDS-PAGE analysis, 52 kDa band for the FAD-binding oxidoreductase-like protein and 49 kDa band for WP_048472121 methyltransferase were detected from the proteins extracted from *E. coli* cell introduced the plasmid pFADO-M2121-I (Supplementary Figure 2). These results indicated that the FAD-binding oxidoreductase-like protein and WP_048472121 are involved in the synthesis of 19:0Me10. Hereafter, we call them the *bfaA* and *bfaB* genes, respectively. In general, the 4-bp overlapping genes are co-transcribed as dicistronic genes in the bacteria, as an example of the ssl2245-sll1130 genes in the cyanobacterium *Synechocystis* sp. PCC 6803 (Srikumar et al., 2017). We speculated that the *bfaAB* genes are an operon in *M. chlorophenolicum.*

**Table 2:** Fatty acid composition of *E. coli* transformants.

	Fatty acid (mol %)							
	Vector control		BfaA^+^		BfaB^+^		BfaAB^+^	
	Exogenous FA 18:1Δ9		Exogenous FA 18:1Δ9		Exogenous FA 18:1Δ9		Exogenous FA 18:1Δ9	
	-	+	-	+	-	+	-	+
14:0	11.7 ± 1.7	5.3 ± 0.2	6.8 ± 0.0	5.2 ± 0.8	6.9 ± 0.3	4.7 ± 0.5	7.9 ± 0.8	5.6 ± 0.2
16:0	42.3 ± 1.6	39.3 ± 1.5	44.2 ± 0.7	38.1 ± 0.0	43.5 ± 0.2	37.0 ± 0.8	44.6 ± 0.4	38.5 ± 2.0
16:1Δ7	–	4.6 ± 0.2	–	4.1 ± 0.1	–	4.2 ± 0.2	–	4.4 ± 0.1
16:1Δ9	3.2 ± 0.5	1.6 ± 0.1	3.6 ± 1.1	1.3 ± 0.1	4.4 ± 0.4	1.5 ± 0.4	3.8 ± 0.4	1.2 ± 0.2
17:1cycloΔ9	22.3 ± 1.1	10.1 ± 0.5	22.5 ± 1.4	11.1 ± 0.3	21.0 ± 0.4	9.3 ± 0.6	21.3 ± 1.0	10.6 ± 0.9
18:0	1.6 ± 0.4	1.2 ± 0.2	1.2 ± 0.1	1.6 ± 0.0	0.96 ± 0.1	2.5 ± 0.9	1.1 ± 0.1	1.4 ± 0.1
18:1Δ9	–	19.8 ± 2.6	–	18.7 ± 1.3	–	23.8 ± 2.1	–	18.0 ± 2.7
18:1Δ11	11.7 ± 1.3	7.1 ± 0.5	14.4 ± 2.4	8.2 ± 0.4	17.6 ± 0.3	10.1 ± 0.5	15.4 ± 1.0	9.2 ± 0.4
19:1cycloΔ9	–	4.8 ± 0.0	–	4.4 ± 0.0	–	3.0 ± 0.5	–	3.6 ± 0.2
19:1cycloΔ11	7.3 ± 1.3	5.6 ± 0.2	7.4 ± 1.4	6.6 ± 0.6	5.7 ± 0.5	4.5 ± 0.5	6.0 ± 0.6	6.1 ± 0.8
19:0Me10	–	–	–	–	–	–	–	0.7 ± 0.0
19:1Me10	–	–	–	–	–	0.5 ± 0.1	–	–

*The fatty acid composition of total lipids from the vector control and BfaA and BfaB-expressing *E. coli* strains. The cells were grown at 37°C for 18 h. As an exogenous fatty acid, 18:1Δ9 (1 mM) was added to the culture. The results were expressed as the mol % of total fatty acids and represent the mean ± standard deviation of three independent experiments. “-”, not detected; 19:1Me10, 10-methylene octadecanoic acid.*

**FIGURE 4:**
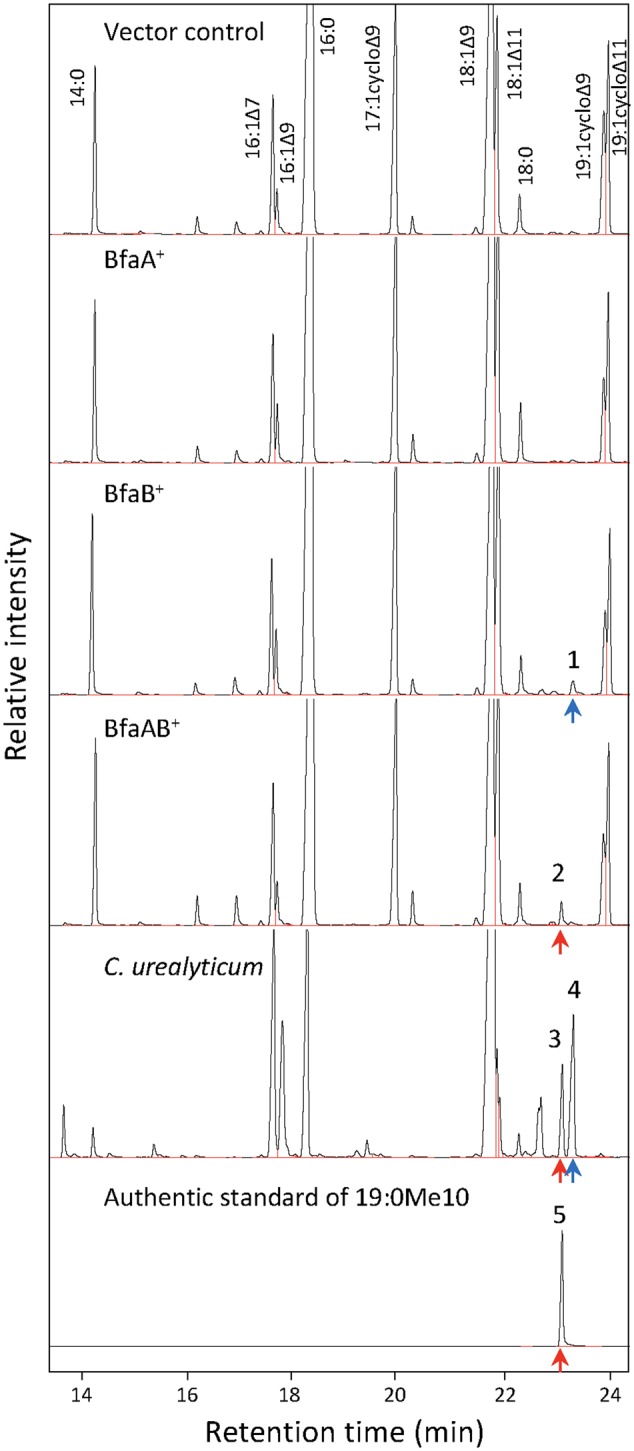
Analysis of fatty acid methylesters by gas chromatography (GC) with a flame-ionization detector. Fatty acids were extracted from *E. coli* and *C. urealyticum* cells, methylesterified, and analyzed by GC-FID. 18:1Δ9 (1 mM) was exogenously added to the culture of *E. coli*. BfaA and BfaB indicate the FAD-binding oxidoreductase and WP_048472121, respectively. Bottom panel indicate the result of the authentic standard of 19:0Me10. Peaks of the identified fatty acid methylesters were indicated as corresponding fatty acids. The retention times of methylesters of 19:0Me10 and 19:1Me10 were indicated by red and blue arrows, respectively. The numbers on the peaks in the Fig are corresponding to the MS analyses shown in **Figure 5**.

**FIGURE 5:**
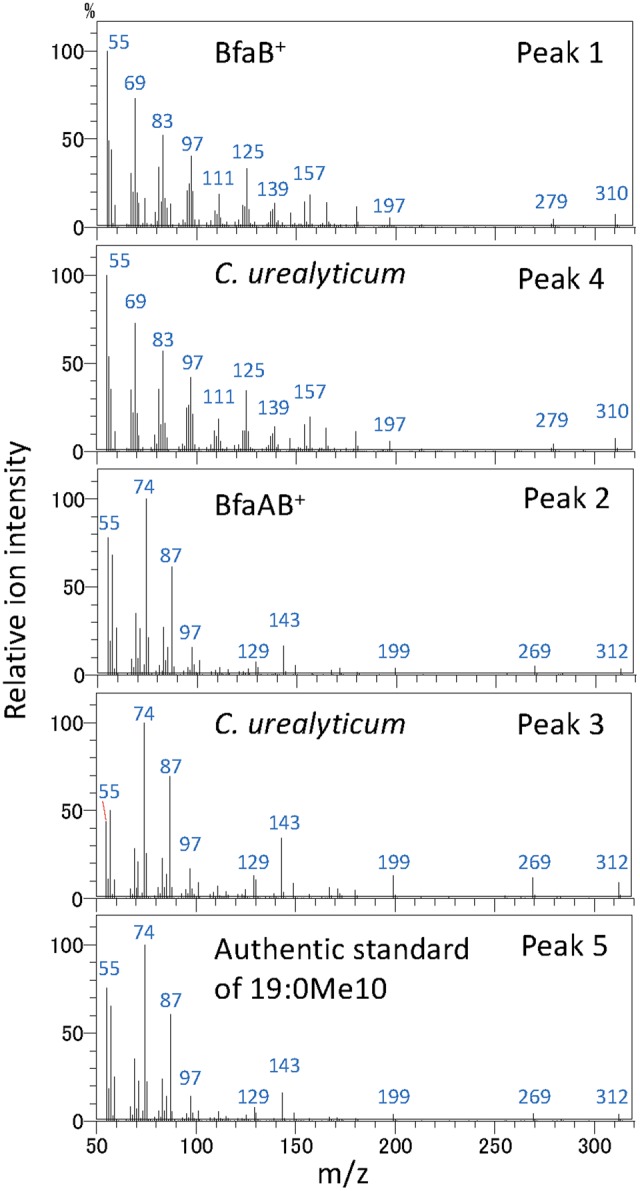
Analysis of fatty acid methylesters by GC with a mass spectrometer. Mass spectra of fatty acid methylester were analyzed by GC with a mass spectrometer. Peak 1, fatty acid extracted from *bfaB*^+^ strain; peak 2, fatty acid extracted from *bfaAB*^+^ strain; peaks 3 and 4, fatty acids extracted from *C. urealyticum* cells; and peak 5, authentic standard of 10-methyl stearic acid. The numbers in this Fig are linked with the peaks in **Figure 4**. BfaA and BfaB indicate FAD-binding oxidoreductase and WP_048472121, respectively.

It has been reported that some *Corynebacterium* species, e.g., ATCC 43042, ATCC 43043, and ATCC 43044, accumulate 10-methylene octadecanoic acid as an intermediate of 19:0Me10 (Couderc et al., 1991). 10-Methylene octadecanoic acid is thought to be produced by the methylation of 18:1Δ9 and then reduced to 19:0Me10. If BfaB and BfaA catalyze the two reaction steps of methylenation and reduction, respectively, then the cells expressing only BfaB should accumulate 10-methylene octadecanoic acid. The results of a fatty acid analysis showed that a peak corresponding to a novel fatty acid was detected in the *E. coli* cells transformed with the *bfaB* gene using the plasmid pM2121 (**Figure 4**). This fatty acid could not be clearly identified as 10-methylene octadecanoic acid, because an authentic standard for this fatty acid was not available and the fragment patterns were also not available in the mass spectrometry data library. However, one of the fatty acid extracted from *C. urealyticum* ATCC 43042 showed same retention time and mass spectra with the characteristic fatty acid extracted from the *E. coli* cell expressing *bfaB* gene (**Figure 4**). The content of the fatty acid in *C. urealyticum* was approximately 3.5% of the total fatty acids as shown in the previous literalities (Couderc et al., 1991). And the mass of the parent ion of this peak was 310, which corresponds to the calculated molecular mass of the methyl ester of 10-methylene octadecanoic acid (**Figure 5**). From these results, we expected that these peaks were for 10-methylene octadecanoic acid.

It has been speculated that the second step of 19:0Me10 biosynthesis includes a reduction reaction using NADPH as a cofactor (Akamatsu and Law, 1970). Our results indicated that BfaA might catalyze the reduction of 10-methylene octadecanoic acid, since this protein is annotated as an “FAD-linked oxidoreductase.” FAD is a cofactor for numerous enzymes that mediate redox reactions. For instance, UDP-*N*-acetylmuramate:NADP^+^ oxidoreductase (MurB) is an FAD-binding enzyme that catalyzes the NADPH-dependent reduction of UDP-*N*-acetyl-3-*O*-(1-carboxyvinyl)-α-D-glucosamine to the corresponding D-lactyl compound, UDP-*N*-acetyl-α-D-muramate. First, NADPH reduces the FAD of the enzyme to NADP^+^, which dissociates from the enzyme. Then, the enzyme interacts with the substrate, UDP-*N*-acetyl-3-*O*-(1-carboxyvinyl)-α-D-glucosamine, and the reduced FAD transfers an electron to the substrate (Fagan and Palfey, 2010). We speculate that a similar reaction mechanism might occur in the case of BfaA.

### Expression of Candidate Assistant Protein

As shown in **Figure 3**, the third conserved gene was located at the upstream of *bfaA* on the opposite coding strand. The orthologs of this gene were found in the same genetic locus on the chromosomes of the organisms categorized into clade B, as shown in **Figure 2**. Genes that adjoin on a chromosome in a head-to-head arrangement are often expressed concomitantly, perhaps due to sharing regulatory elements. Furthermore, the products of such genes may function together. The upstream gene was annotated to encode a homolog of polyketide cyclase, which has a “hydrophobic ligand binding site” and may interact with the hydrocarbon chains containing carboxyl or hydroxyl groups. This genomic information may indicate that this gene has the potential of assistant for biosynthesis of 19:0Me10 and the expression of this gene improve the productivity of 19:0Me10. To evaluate the possibility that the third gene product is involved in the formation of 19:0Me10, we expressed the gene for polyketide cyclase together with the *bfaA* and *bfaB* genes in *E. coli* cells using the plasmid pFADO-M2121-PCYC (Supplementary Figure 1). However, the abundance of mBFA in the cells was not altered compared with that in the cells expressing the *bfaA* and *bfaB* genes (data not shown), indicating that the third gene located upstream of the *bfaA* and *bfaB* genes does not seem to be an essential factor for mBFA synthesis or does not function in *E. coli* cells.

### Substrate Specificity of BfaA and BfaB

*Escherichia coli* cells synthesize the unsaturated fatty acids 16:1Δ9 and 18:1Δ11 as components of their membrane lipids, but they do not synthesize 18:1Δ9, 18:2Δ9,12, or 18:3Δ6,9,12. In general, fatty acid-modifying enzymes, including the acyl-lipid fatty acid desaturases, react with the fatty acids in a position-specific manner (Los and Murata, 1998). We also reported that the cyclopropane fatty acid synthase in *E. coli* introduced a cyclopropane ring into the natural substrates 16:1Δ9 and 18:1Δ11, and the artificial substrates 18:1Δ9, 18:2Δ9,12, and 18:3Δ9,12,15, but not 18:3Δ6,9,12 (Machida et al., 2016). According to the results in **Table 1**, BfaA and BfaB specifically react with 18:1Δ9, but not 16:1Δ9 and 18:1Δ11, in *E. coli*. It was unclear whether BfaA and BfaB introduce a branched chain at the Δ9 position or other positions in various unsaturated fatty acids. We analyzed the fatty acid compositions of the vector control and BfaA and BfaB-transformed strains cultivated in liquid media supplemented with 1 mM each of 16:1Δ9, 18:1Δ11, 18:2Δ9,12, 18:3Δ9,12,15, and 18:3Δ6,9.12 as exogenous fatty acids. The results showed that the fatty acid composition of the BfaA and BfaB-transformed strains was not different from that of the vector control strain and no novel mBFAs were detected (Supplementary Table 3). These results suggest that BfaA and BfaB can specifically modify only the Δ9 position of the C18 mono-unsaturated fatty acid, 18:1Δ9, as a substrate for the production of mBFA. *M. chlorophenolicum* has 16:1Δ9 and 18:1Δ9 as unsaturated fatty acids and synthesizes 19:0Me10 (Hagglblom et al., 1994). Based on these phenomena, it is concluded that 18:1Δ9 produced by the desaturation of 18:0 might be converted to 19:0Me10 by BfaA and BfaB.

### Homologous Proteins of the FAD-binding Oxidoreductase and the SAM-Dependent Methyltransferase in the Organisms Producing mBFA

From our current study, it was suggested that BfaA and BfaB encoding the FAD-binding oxidoreductase and the SAM-dependent methyltransferase, respectively, are involved in the synthesis of 19:0Me10 in *M. chlorophenolicum*. As shown in **Figure 2**, the paralogs of the *bfaA* and *bfaB* operon were conserved in other bacteria producing 19:0Me10 and similarities of paralogs to BfaA and BfaB were more than 50%, except *R. phenolicus* DSM 44812. In this organism, the genes encoding the most similar enzymes for BfaA and BfaB, i.e., WP_068165945 and WP_068165842, were separately located in the chromosome and the similarity of these proteins to the other homologs were rather low in **Figure 2**. We, then, compared the primary sequences of the homologous proteins of BfaA and BfaB in these bacteria except *R. phenolicus* DSM 44812 (Supplementary Figures 3A,B). These proteins were very similar to each other, although the FAD-binding oxidoreductase and the SAM-dependent methyltransferase from *C. urealyticum* ATCC 43042 possess the unique short-inserted sequences of 11 and 19 amino acid residues at 398 and 176 amino acids, respectively. According to the previous publications, only from *C. urealyticum* ATCC 43042, 10-methylene octadecanoic acid is detected as the intermediate of 19:0Me10 synthesis (Couderc et al., 1991). The unique extensions in the enzymes in *C. urealyticum* ATCC 43042 may extremely enhance the activity of the SAM-dependent methyltransferase producing 10-methylene octadecanoic acid and/or significantly decrease the activity of the FAD-binding oxidoreductase processing 10-methylene octadecanoic acid to 19:0Me10, and then 10-methylene octadecanoic acid might be accumulated.

## Conclusion

We successfully synthesized 19:0Me10 in *E. coli* by the heterologous expression of BfaA and BfaB from *M. chlorophenolicum*. Cells expressing *bfaB* alone might only produce 10-methylene octadecanoic acid as an intermediate of 19:0Me10 production. It seems that an FAD-linked oxidoreductase encoded by *bfaA* contributes to the reduction reaction that is required for the conversion of 10-methylene octadecanoic acid to 19:0Me10. Our assay to determine the substrate specificity of BfaA and BfaB indicated that only 18:1Δ9 is used as a substrate. We expect that these findings of a novel 19:0Me10 biosynthetic system will be helpful in healthcare or industry.

## Author Contributions

SM substantially performed most of the study with the consultation by IS and RB contributed GC–MS analyses. SM and IS summarized all the data and described manuscript.

## Conflict of Interest Statement

We have submitted a patent including the method to produce 10-methyl stearic acid using the genes identified in this manuscript. The authors declare that the research was conducted in the absence of any commercial or financial relationships that could be construed as a potential conflict of interest.

## Footnotes

1 http://www.jcm.riken.jp/cgi-bin/jcm/jcm_grmd?GRMD=26

2 www.ncbi.nlm.nih.gov/pubmed

3 http://www.clustal.org/

4 http://tree.bio.ed.ac.uk/software/figtree/

5 http://www.genome.jp/tools-bin/clustalw

6 http://www.ch.embnet.org/software/BOX_form.html
