# Supplementary material for: Expression of Genes for a Flavin Adenine Dinucleotide-Binding Oxidoreductase and a Methyltransferase from Mycobacterium chlorophenolicum Is Necessary for Biosynthesis of 10-Methyl Stearic Acid from Oleic Acid in Escherichia coli
Source: Front Microbiol. 2017 Oct 23;8:2061. doi: 10.3389/fmicb.2017.02061 (PMC5660069; doi:10.3389/fmicb.2017.02061)
Supplement: Supplementary file 1 [file Table_1.docx]

**Supplementary Table 1**

Plasmids used in this study

| Plasmids | Description | Reference |
| --- | --- | --- |
| pTCHT2031V | Carrying *trc* promoter, Amp^r^, and Cm^r^ | Ishizuka et al., 2006 |
| pTHT2031V | Carrying *trc* promoter, and Amp^r^ | This study |
| pAM1146 | Carrying Amp^r^, and Sp^r^ | Tsinoremas et al., 1994 |
| pMD-M1942 | pMD19 with WP_048471942, carrying Amp^r^ | This study |
| pMD-M2121 | pMD19 with WP_048472121, carrying Amp^r^ | This study |
| pMD-FADO-4-M2121 | pMD19 with WP_048472021 and WP_048472121 with overlap of 4 base pair, carrying Amp^r^ | This study |
| pMD-M1942S | pMD-M1942 with Sp^r^ | This study |
| pMD-M2121S | pMD-M2121 with Sp^r^ | This study |
| pMD-FADO-4-M2121S | pMD-FADO-4-M2121 with Sp^r^ | This study |
| pM1942 | Carrying *trc* promoter, WP_048471942, Sp^r^, and Amp^r^ | This study |
| pM2121 | Carrying *trc* promoter, WP_048472121, Sp^r^, and Amp^r^ | This study |
| pFADO-4-M2121 | Carrying *trc* promoter, WP_048472021 and WP_048472121 with overlap of 4 base pair, Sp^r^, and Amp^r^ | This study |
| pFADO-M2121-I | pFADO-4-M2121 with insertion of SD sequence between WP_048472021 and WP_048472121 | This study |
| pFADO-M2121-S | pFADO-4-M2121 with substitution of SD sequence between WP_048472021 and WP_048472121 | This study |
| pFADO-M2121-PCYC | pFADO-M2121-I with PCYC | This study |
| pTAKN-2-UmaA | pTAKN-2 with UmaA, carrying Kan^r^ | Artificially synthesized |
| pTAKN-2-UmaAS | pTAKN-2-UmaA with Sp^r^ | This study |
| pUmaA | Carrying *trc* promoter, UmaA, Sp^r^, and Amp^r^ | This study |
