# Supplementary material for: Expression of Genes for a Flavin Adenine Dinucleotide-Binding Oxidoreductase and a Methyltransferase from Mycobacterium chlorophenolicum Is Necessary for Biosynthesis of 10-Methyl Stearic Acid from Oleic Acid in Escherichia coli
Source: Front Microbiol. 2017 Oct 23;8:2061. doi: 10.3389/fmicb.2017.02061 (PMC5660069; doi:10.3389/fmicb.2017.02061)
Supplement: Supplementary file 2 [file Table_2.docx]

**Supplementary Table 2**

Primers used in this study

| No. | Name | Sequence (5′-3′) |
| --- | --- | --- |
| 1 | pTCHT_Cm_remove_InF_F | TTTTTGCTTCATCGCTTAAGGCAGTTATTG |
| 2 | pTCHT_Cm_remove_InF_R | ACTGCCTTAAAAAAAGAAGCAAAAAGCCTA |
| 3 | M1942_Nde_F | CATATGATGACGACCGCTGAAACGG |
| 4 | M1942_Bam_R | GGATCCTCACGATTTCGATGCCCGCG |
| 5 | M2121_Nde_F | CATATGATGACGACTTTTCGGGAACG |
| 6 | M2121_Bam_R | GGATCCTCAGGCGGTCCACCAAGGCC |
| 7 | FAD_Nde_F | CATATGATGTCTGTTCCCGCAACCGA |
| 8 | Sp_Bgl_F | GGAGATCTATCAATTCCCCTGCTCGCGC |
| 9 | Sp_Bam_R | GGGGATCCTCCCAATTTGTGTAGGGCTT |
| 10 | SD_add_S_F | AGGAGACAATGACGACTTTTCGGG |
| 11 | SD_add_S_R | TCGTCATTGTCTCCTTACCGCCTTCGCATACAGG |
| 12 | SD_add_I_F | AGGAGGAATAAACCATGACGACTTTTCGGGAACGACCG |
| 13 | SD_add_I_R | TGGTTTATTCCTCCTTCATTGTCGCCGTACCGCC |
| 14 | PCYC_inf_F | TGGTGGACCGCCTGAGAGGAGTTCGCACCGATGGG |
| 15 | PCYC_inf_R | AGCAGGGGAATTGATCTACACGTCCTGGACGGCCTC |
| 16 | Sp_Bam_F | GGGGATCCATCAATTCCCCTGCTCGCGC |
| 17 | Sp_Bgl_R | GGAGATCTTCCCAATTTGTGTAGGGCTT |
