# Supplementary material for: Expression of Genes for a Flavin Adenine Dinucleotide-Binding Oxidoreductase and a Methyltransferase from Mycobacterium chlorophenolicum Is Necessary for Biosynthesis of 10-Methyl Stearic Acid from Oleic Acid in Escherichia coli
Source: Front Microbiol. 2017 Oct 23;8:2061. doi: 10.3389/fmicb.2017.02061 (PMC5660069; doi:10.3389/fmicb.2017.02061)
Supplement: Supplementary file 3 [file Table_3.docx]

**Supplementary Table 3. Fatty acid composition of *E. coli* transformants with various fatty acids**

As exogenous fatty acids, 16:1Δ9, 18:1Δ11, 18:2Δ9,12, 18:3Δ9,12,15, and 18:3Δ6,9,12 (1 mM) were added to the culture. The results were expressed as the mol % of total fatty acids and represent the mean ± standard deviation of three independent experiments. “-”, not detected.

|  | Fatty acid (mol %) | | | | | | | | | | |
| --- | --- | --- | --- | --- | --- | --- | --- | --- | --- | --- | --- |
|  | Vector control | | | | |  | BfaAB^+^ | | | | |
|  | Exogenous FA | | | | |  | Exogenous FA | | | | |
|  | 16:1Δ9 | 18:1Δ11 | 18:2Δ9,12 | 18:3Δ9,12,15 | 18:3Δ6,9,12 |  | 16:1Δ9 | 18:1Δ11 | 18:2Δ9,12 | 18:3Δ9,12,15 | 18:3Δ6,9,12 |
| 14:0 | 5.5 ± 0.4 | 5.4 ± 0.3 | 8.4 ± 2.1 | 8.3 ± 2.1 | 5.2 ± 1.7 |  | 4.5 ± 0.7 | 5.6 ± 0.3 | 8.1 ± 1.9 | 7.8 ± 1.4 | 5.1 ± 1.4 |
| 16:0 | 42.0 ± 1.6 | 36.1 ± 1.0 | 36.6 ± 0.9 | 38.5 ± 1.9 | 39.3 ± 0.2 |  | 40.3 ± 1.4 | 34.7 ± 1.3 | 37.3 ± 2.3 | 38.6 ± 1.1 | 40.1 ± 0.4 |
| 16:1Δ9 | 7.6 ± 1.8 | 4.5 ± 0.3 | 5.2 ± 1.3 | 3.2 ± 1.1 | 6.2 ± 1.0 |  | 8.1 ± 0.9 | 4.6 ± 0.3 | 5.6 ± 1.2 | 3.0 ± 1.0 | 6.3 ± 1.1 |
| 16:2Δ7,10 | - | - | 4.4 ± 1.6 | - | - |  | - | - | 4.1 ± 1.4 | - | - |
| 16:3Δ7,10,13 | - | - | - | 4.9 ± 0.3 | - |  | - | - | - | 4.8 ± 0.2 | - |
| 16:3Δ4,7,10 | - | - | - | - | 2.8 ± 0.7 |  | - | - | - | - | 2.7 ± 0.8 |
| 17:1cycloΔ9 | 24.9 ± 1.9 | 15.3 ± 0.6 | 14.4 ± 2.3 | 9.2 ± 3.8 | 17.2 ± 2.6 |  | 23.8 ± 0.9 | 14.8 ± 0.4 | 14.3 ± 1.8 | 9.3 ± 3.8 | 16.4 ± 1.9 |
| 18:0 | 1.0 ± 0.2 | 0.9 ± 0.1 | 3.4 ± 0.6 | 1.9 ± 0.5 | 1.9 ± 0.7 |  | 1.0 ± 0.1 | 0.9 ± 0.2 | 3.5 ± 0.7 | 1.8 ± 0.4 | 1.7 ± 0.7 |
| 18:1Δ11 | 15.4 ± 1.4 | 29.3 ± 1.6 | 11.4 ± 2.3 | 11.9 ± 1.1 | 14.1 ± 0.5 |  | 18.9 ± 1.8 | 32.0 ± 0.9 | 11.6 ± 2.8 | 12.3 ± 1.3 | 13.8 ± 0.6 |
| 18:2Δ9,12 | - | - | 11.5 ± 1.0 | - | - |  | - | - | 11.3 ± 0.8 | - | - |
| 18:3Δ9,12,15 | - | - | - | 13.9 ± 0.7 | - |  | - | - | - | 14.9 ± 0.8 | - |
| 18:3Δ6,9,12 | - | - | - | - | 11.8 ± 1.2 |  | - | - | - | - | 11.1 ± 1.0 |
| 19:1cycloΔ11 | 3.6 ± 0.8 | 8.5 ± 0.8 | 4.1 ± 2.1 | 6.1 ± 1.8 | 2.3 ± 1.0 |  | 3.4 ± 0.6 | 7.4 ± 0.6 | 3.8 ± 1.9 | 5.7 ± 2.3 | 2.2 ± 0.9 |
| 19:2Δ12cycloΔ9 | - | - | 0.9 ± 0.3 | - | - |  | - | - | 0.8 ± 0.3 | - | - |
| 19:3Δ12,15cycloΔ9 | - | - | - | 2.2 ± 1.1 | - |  | - | - | - | 2.0 ± 0.9 | - |
| mBFAs | - | - | - | - | - |  | - | - | - | - | - |
