# Supplementary material for: Expression of Genes for a Flavin Adenine Dinucleotide-Binding Oxidoreductase and a Methyltransferase from Mycobacterium chlorophenolicum Is Necessary for Biosynthesis of 10-Methyl Stearic Acid from Oleic Acid in Escherichia coli
Source: Front Microbiol. 2017 Oct 23;8:2061. doi: 10.3389/fmicb.2017.02061 (PMC5660069; doi:10.3389/fmicb.2017.02061)
Supplement: Supplementary file 4 [file Image_1.pdf]

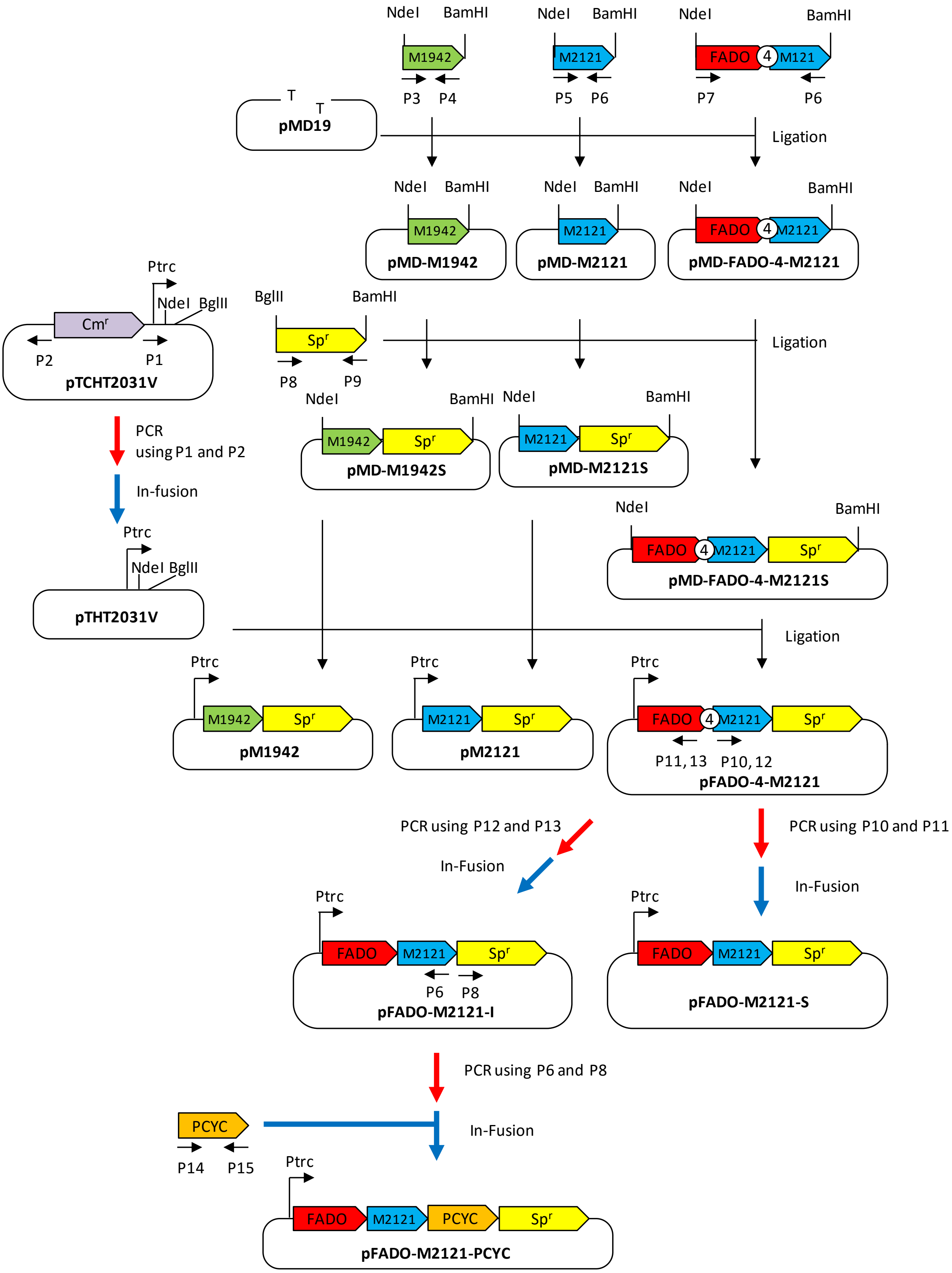

NdeI
BamHI
M1942
P3
P4
M2121
P5
P6
FADO
4
M121
P7
T
pMD19
Ligation
pMD-M1942
pMD-M2121
M2121
pMD-FADO-4-M2121
Ptrc
BglII
Cmʳ
P2
P1
pTCHT2031V
Spʳ
P8
P9
pMD-M1942S
pMD-M2121S
PCR
using P1 and P2
In-fusion
pMD-FADO-4-M2121S
pTHT2031V
pM1942
pM2121
P11, 13
P10, 12
pFADO-4-M2121
PCR using P12 and P13
In-Fusion
PCR using P10 and P11
pFADO-M2121-I
pFADO-M2121-S
PCR using P6 and P8
PCYC
P14
P15
pFADO-M2121-PCYC

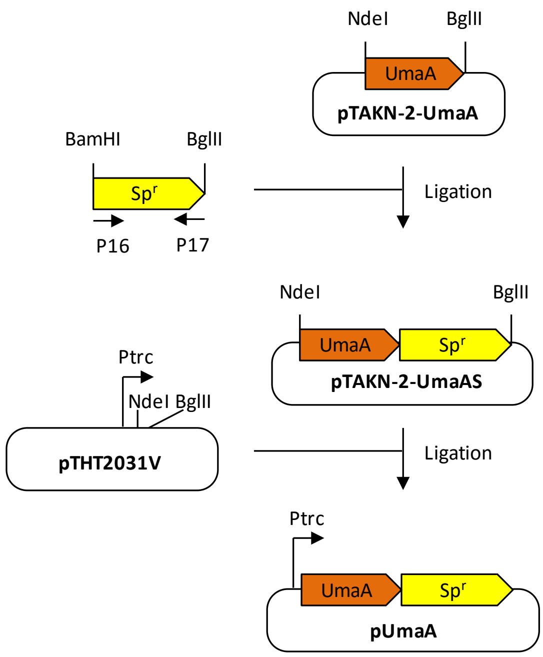

Supplementary Figure 1. Construction of the plasmid

Scheme of the engineered DNA fragment for expression in *E. coli*. The fragments encoding WP_048471942, WP_048472121, flavin adenine dinucleotide (FAD)-binding oxidoreductase, and polyketide cyclase were amplified from *Mycobacterium chlorophenolicum* and the target genes were expressed under the control of the *trc* promoter. M1942, WP_048471942; M2121, WP_0484712121 (BfaB); FADO, FAD-binding oxidoreductase; PCYC, polyketide cyclase. The number "4" in the circle indicates the overlap of 4 base pairs between the two tandem genes.
