# Supplementary material for: Expression of Genes for a Flavin Adenine Dinucleotide-Binding Oxidoreductase and a Methyltransferase from Mycobacterium chlorophenolicum Is Necessary for Biosynthesis of 10-Methyl Stearic Acid from Oleic Acid in Escherichia coli
Source: Front Microbiol. 2017 Oct 23;8:2061. doi: 10.3389/fmicb.2017.02061 (PMC5660069; doi:10.3389/fmicb.2017.02061)
Supplement: Supplementary file 5 [file Image_2.pdf]

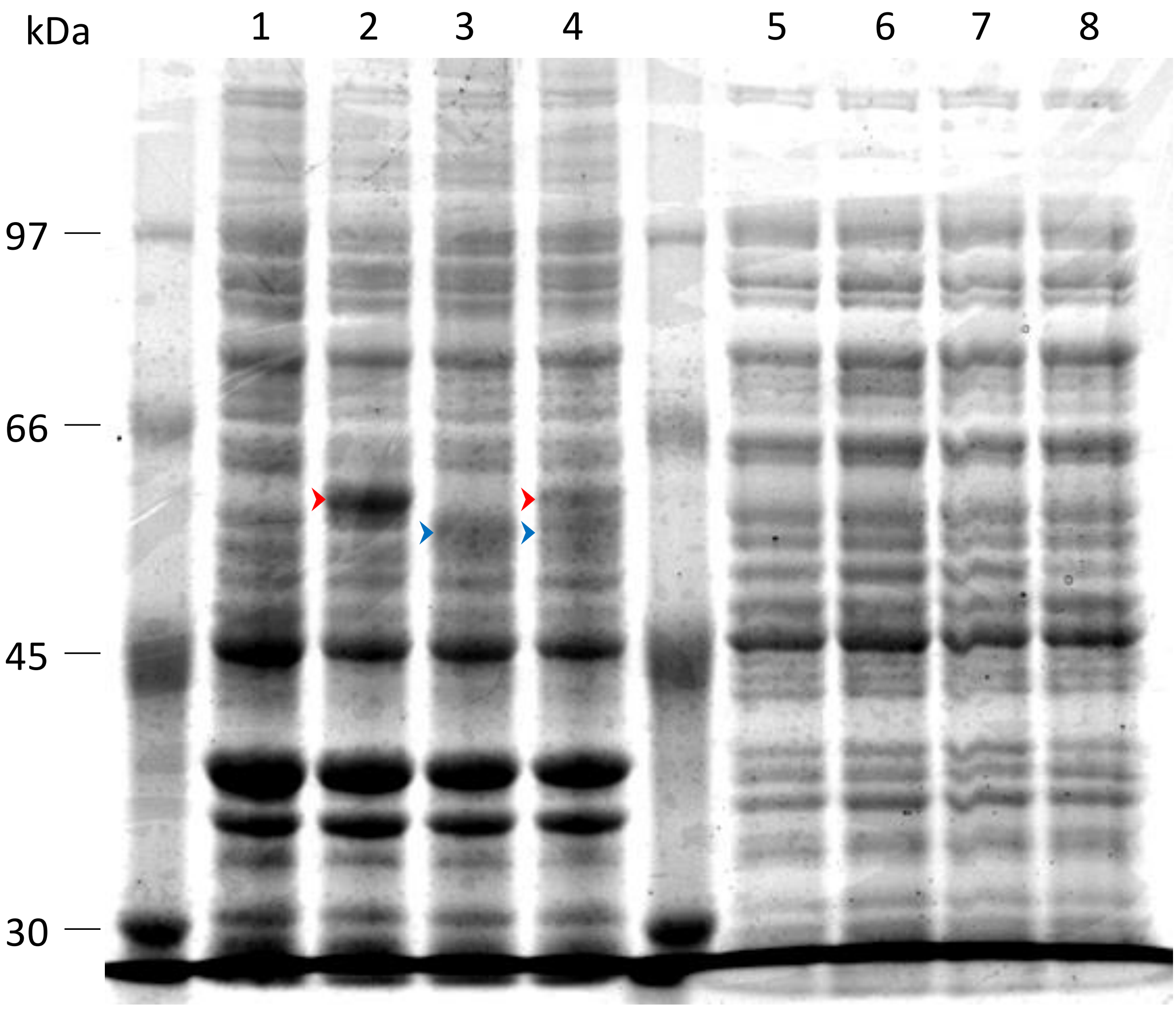

Supplementary Figure 2. SDS-PAGE analysis of the *E. coli* cells

Proteins extracted from the *E. coli* cells were used for SDS-PAGE and stained with Coomassie Brilliant Blue R-250. Red and blue arrows indicate estimated band for BfaA (52kDa) and BfaB (49kDa), respectively. Lane 1, insoluble fraction of vector control strain; Lane 2, insoluble fraction of *bfaA*$^{+}$ strain; Lane 3, insoluble fraction of *bfaB*$^{+}$ strain; Lane 4, insoluble fraction of *bfaAB*$^{+}$ strain; Lane 5, soluble fraction of vector control strain; Lane 6, soluble fraction of *bfaA*$^{+}$ strain; Lane 7, soluble fraction of *bfaB*$^{+}$ strain; Lane 8, soluble fraction of *bfaAB*$^{+}$ strain.
