# Supplementary material for: Expression of Genes for a Flavin Adenine Dinucleotide-Binding Oxidoreductase and a Methyltransferase from Mycobacterium chlorophenolicum Is Necessary for Biosynthesis of 10-Methyl Stearic Acid from Oleic Acid in Escherichia coli
Source: Front Microbiol. 2017 Oct 23;8:2061. doi: 10.3389/fmicb.2017.02061 (PMC5660069; doi:10.3389/fmicb.2017.02061)
Supplement: Supplementary file 6 [file Image_3.pdf]

A. FAD-binding oxidoreductases

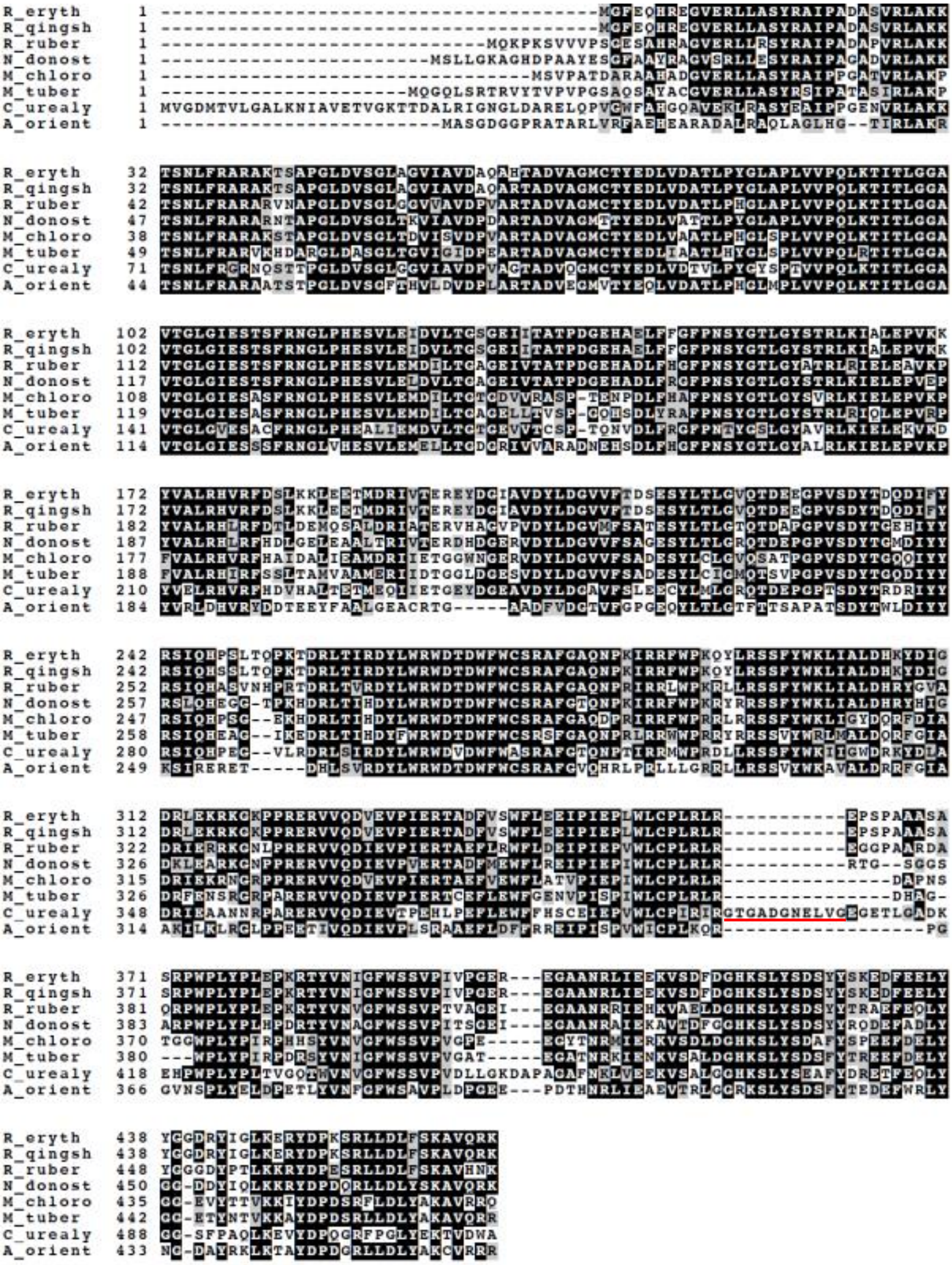

B. SAM-dependent methyltransferases

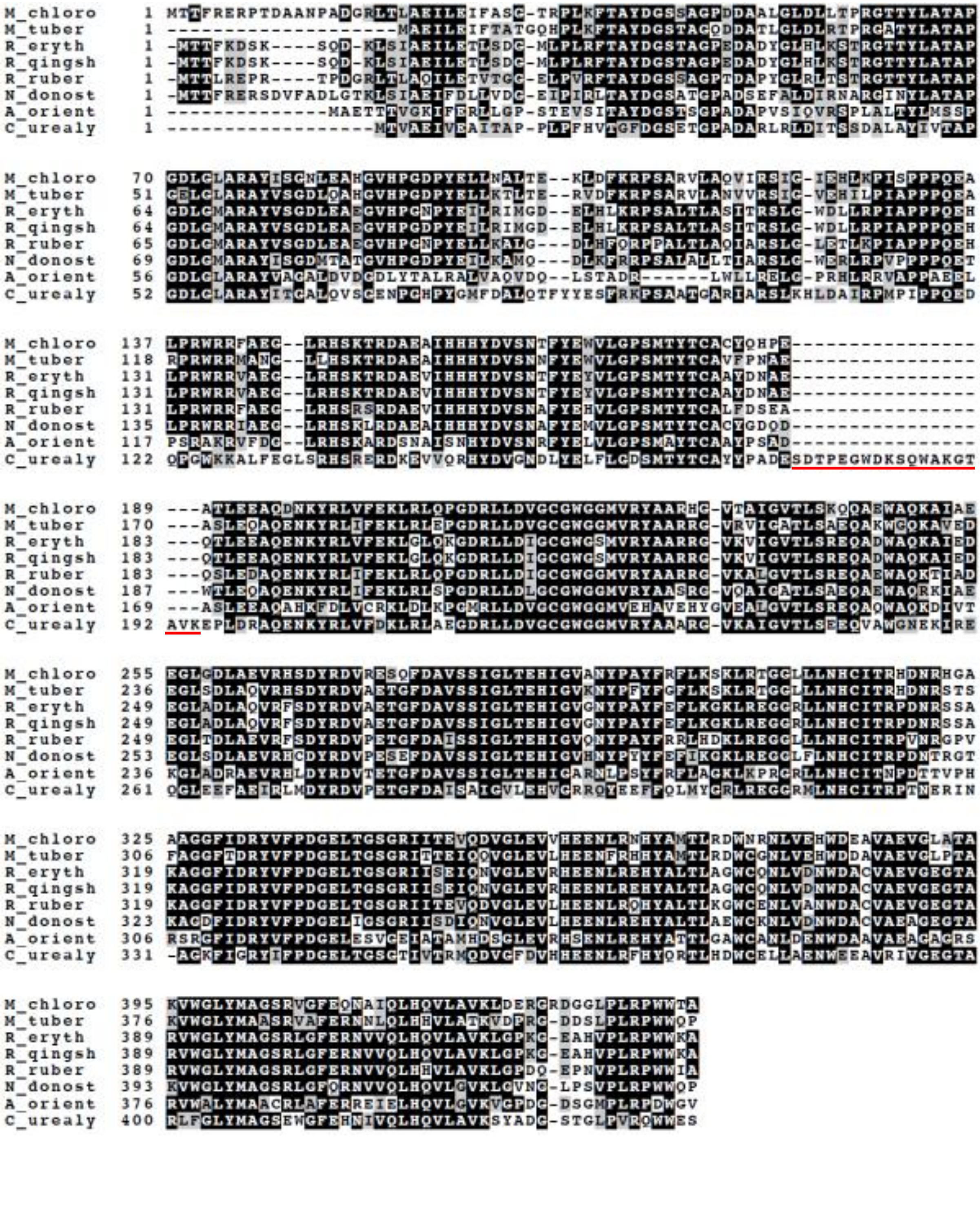

Supplementary Figure 3. Alignments of amino acid sequences of homologous proteins of FAD-binding oxidoreductase WP_048472120 and SAM-dependent methyltransferase WP_048472121

A. Amino acid sequences of homologous proteins of FAD-binding oxidoreductase WP_048472120. R_eryth, WP_019746543 of *Rhodococcus erythropolis* R138; R_qingsh, WP_007731548 of *Rhodococcus qingshengii* BKS 20-40; R_ruber, WP_003935378 of *Rhodococcus ruber* BKS 20-38; N_donost, WP_077118793 of *Nocardia donostiensis* X 1654; M_chloro, WP_048472120 of *Mycobacterium chlorophenolicum* JCM 7439; M_tuber, WP_003917236 of *Mycobacterium tuberculosis* H37Rv; C_urealy, WP_012359407 of *Corynebacterium urealyticum* ATCC 43042; A_orient, WP_037303122 of *Amycolatopsis orientalis* DSM 40040. **B.** Amino acid sequences of homologous proteins of SAM-dependent methyltransferase WP_048472121. M_chloro, WP_048472121 of *Mycobacterium chlorophenolicum* JCM 7439; M_tuber, WP_003420415 of *Mycobacterium tuberculosis* H37Rv; R_eryth, WP_019746542 of *Rhodococcus erythropolis* R138; R_qingsh, WP_003943932 of *Rhodococcus qingshengii* BKS 20-40; R_ruber, WP_003935379 of *Rhodococcus ruber* BKS 20-38; N_donost, WP_077118797 of *Nocardia donostiensis* X 1654; A_orient, WP_037303124 of *Amycolatopsis orientalis* DSM 40040; C_urealy, WP_012359408 of *Corynebacterium urealyticum* ATCC 43042. Inserted sequences in *C. urealyticum* ATCC 43042 were indicated by red underlines.
